# The Politicised Child During the Seventeenth-Century British Civil Wars: An Historical Perspective on Representations of Children and Trauma During Conflict

**DOI:** 10.1007/s11013-021-09741-6

**Published:** 2021-08-18

**Authors:** Ismini Pells

**Affiliations:** University of Leicester, Leicester, UK

**Keywords:** Children, Warfare, Trauma, Britain, Early modern history

## Abstract

The seventeenth-century British Civil Wars had a scale and impact to rival modern conflicts and its effects extended to children as well as adults. What might be today termed “child soldiers” were found in the armies in combat and supporting roles. Many more were witnesses to the conflict or had their lives changed by its consequences. This article is an historical case study of socio-cultural constructions of children, childhood and warfare. It aims to highlight the diverse nature of both historic and modern child experiences of warfare, and the plethora of ways that these experiences were and are understood and represented by adults. It argues that the evidence from the Civil Wars supports the scholarship of child psychologists such as Derek Summerfield that children in conflict should not always be regarded as victims but could display agency, whilst also acknowledging social, cultural, economic and political pressures. Although children in the Civil Wars may have experienced trauma, the evidence is insufficient to prove this and evidence for a contemporary concept of the psychologically damaged child as a result of conflict is ambiguous. However, what the evidence does uncover is the ways in which adults used representations of children to express their own anxieties about the Civil Wars.

## Introduction

The British Civil Wars, fought between 1642 and 1651, were based on a dispute between the king and parliament over the extent of their respective powers in the government of England. The dispute intermeshed with a wider series of conflicts that involved Scotland and Ireland, as well as England and Wales. They resulted in the execution of King Charles I in 1649, and the establishment of republican government throughout the British Isles, before the Restoration of Charles II to the throne in 1660. The British Civil Wars make an ideal case study for an historical perspective on the effects of armed conflict on children. A vast proportion of the population was mobilized for the various armies, perhaps as many as one in five adult males in England alone, whilst recent estimates suggest that across the British Isles a greater percentage of the population died as a result of the Civil Wars than both World Wars (Donagan 2008:216; Gentles 2007:436–437). Few regions were untouched by the fighting, whilst the Civil Wars continued to maintain a prominent place in folk traditions well into the nineteenth century (Stoyle 2003:204–205). The scale and impact of the conflict meant that, as this article will show, many children became involved in the armies that fought on the royalist and parliamentarian sides and were subject to both the direct and indirect consequences of the war.

Studying the impact of armed conflict on children from an historical perspective is a valuable exercise. It acts as a reminder that there is no agreed concept of what constitutes a child, that is, the “embodied individual defined as nonadult” (Gittins 2009:37). Even in the early modern period, writers were preoccupied and confounded by how to define a child (French 2019:3). Similarly, scholars have long debated the concept of childhood (the status accorded to those defined as a non-adult), which is now agreed to be “always lived and defined in cultural and economic contexts” (Gittins 2009:37; Davin 1999:15). It has been said that childhood is an adult construction imbued with “cultural representations that serve to disguise difference between children—whether terms of gender, ethnicity, class or physical ability” (Gittins 2009:36–37). That said, this viewpoint should not be accepted without three qualifications. Firstly, childhood is not merely determined by adults but as William Corsaro contended, children “negotiate, share and create culture with adults and each other” (Wyness 2012:25). Secondly, even if adults might be in a stronger position than children to impose their ideals and definitions of childhood, adults often have competing interests and thus differing definitions amongst themselves (Davin 1999:19, 33). Thirdly, economic and material factors, such as living in poverty, also have an impact on childhood, which should not be neglected. These factors may not be universally experienced or communicated in a universal language, but it may be speculated that they produce certain worldviews (Wyness 2012:23).

Accessing early modern children’s voices is an almost impossible task, not least due to the scant survival of sources. Memoirs and autobiographies might contain snippets from the author’s youth but “memory is a slippery fish and operates often simultaneously at different levels, arguably being reconstructed over time” (Gittins 2009:36). Children’s speeches and activities were sometimes recorded at the time of their occurrence by adults in diaries, legal records or didactic publications, but these were chronicled according to the adults’ own perceptions and for their own purposes (Fletcher and Hussey 1999:1, 4–5). Nevertheless, whilst historians must be cautious of treating adults’ records of children’s utterances and activities as exact representations, these may still reveal how people reacted to, and made sense of, experiences (Fletcher and Hussey 1999:11). As Hugh Cunningham maintained, attitudes towards children recorded in personal, legal and didactic texts were often taken seriously, reflected common practice and shaped adults’ behaviour towards children. These will have had an impact on the way childhood was experienced, along with the other economic, demographic and political structures that shape society (Cunningham 2005:3).

Any historical enquiry into the role of children and armed conflict in the emergence and evolution of theories of human psyche and psychological trauma must start by acknowledging the numerous methodological problems with identifying and analysing psychological issues in the past. As Jason Crowley has observed, the belief that “susceptibility to posttraumatic stress disorder/combat stress injury is diachronically universal is slowly gaining ground” (Crowley 2014:105). The argument from universality began with reference to the Ancient Greeks, with scholars such as the psychiatrist Jonathan Shay and classicist Lawrence Tritle, but has since filtered out into diverse historical periods and regions (Shay 1995, 2002; Tritle 2000). Such a view, as Crowley pointed out, rests on “an implicit belief in historically transcendental human equivalence, that is to say, that since modern humans are the equivalent of ancient humans, they are not only both equally susceptible to PTSD, but the presence or absence thereof can be detected by the same diagnostic criteria.” As such, “it fails to recognize that PTSD/CSI results from the interaction of two variables, namely the human being and his or her environment,” which “is critical, of course, because neither variable is historical transcendental.” The attitudes and core beliefs adopted by humans change, as does the sociomilitary environment in which the fighting/violence takes place. Consequently, “This naturally allows for the possibility that although the modern combatant and his sociomilitary environment combine to produce a susceptibility to PTSD/CSI, a very different historically specific combination could just as easily reduce, suppress, or even eliminate that susceptibility” (Crowley 2014:105–106).

Crowley’s arguments may be regarded as the apogee of scholarly cynicism on the presence of trauma in the past but even these allow for the possibility that exposure to conflict in the past produced emotional, perhaps lasting psychological, responses, albeit these may be unrecognizable to scholars in the present. Crowley’s criticisms of universality make an important contribution when considering what the past might contribute towards the debates surrounding the psychological impact of conflict on children in the present. Child psychologists such Derek Summerfield and Patrick Bracken have protested against the tendency in modern society to reduce complex experiences to the single category of “trauma” and a failure to distinguish between subjective distress and objective disorder (Summerfield 2000:423, 427; Bracken 2002:4). Summerfield contended that “Every culture has its own reserves of psychological knowledge, range of attributions to assign to adverse experience, and forms of accommodation, struggle and help-seeking” (Summerfield 2000:421–422). Despite this, Western psychological frameworks and practices are seen “as essentially having universal validity” and characterisations of childhood trauma are based on a Western vision of childhood that may not be applicable to all parts of the world (Summerfield 2000:421, 427). To this I might add that such characterisations may not also be applicable to all times. Didier Fassin and Richard Rechtman charted what they deemed to be the “invention of post-traumatic stress in the late nineteenth century” and “its rediscovery in the late twentieth century”. They neither doubted that individuals in the past who experienced a dramatic event suffered psychologically nor did they question the variations in the ways trauma is adopted in multiple cultural contexts today. Instead, Fassin and Rechtman explored the process whereby the reality of psychological suffering has come to be identified, legitimized and receive social recognition using a particular language based on the “condition of victimhood” (Fassin and Rechtman 2009:5–9).

This article will demonstrate the limitations of characterisations of children in conflict that are based on understandings of trauma grounded in the “condition of victimhood” when uncovering the diverse and complex nature of childhood experiences of conflict in the past. Taking the British Civil Wars as an historical case study, I will argue that the evidence supports Summerfield’s arguments that children in conflict should not entirely be regarded as unwilling or helpless victims. They were capable at least in some cases of displaying a sense of agency and even pride in their actions, even if they were simultaneously subjected to social, cultural, economic or political pressures. I will take the stance that whilst it cannot be ruled out that children in the Civil Wars experienced psychological trauma as a result of their experiences, the evidence is insufficient to prove this. Contemporaries certainly viewed children as impressionable and vulnerable to adverse psychological conditions. They also took steps to protect children during warfare, even if these were sometimes disregarded. But they appear to have devoted little consideration to if or how exposure to conflict might cause lasting psychological damage to children. As well as reinforcing the dangers of the presumed universality of understandings of “childhood” or “trauma”, this article will draw attention to the fact that representations of children as victims in warfare often convey more about adult agendas than actual childhood experiences. Historical sources, in which children’s own voices are either absent or mitigated by hindsight and adult perspectives, are especially useful in exploring this phenomenon. There is enough evidence from the British Civil Wars to suggest that children frequently faced violence and cruelties, but I maintain that the precise ways in which this was reported is most revealing of contemporary adults’ own anxieties about the conflict.

## “Child Soldiers” in the British Civil Wars

UNICEF defines a “child soldier” as “any child, boy or girl, under the age of 18, who is part of any kind of regular or irregular armed force or armed group in any capacity,” a definition which “does not only refer to a child who is carrying, or has carried, weapons” (UNICEF 1997). What might thus be termed today “child soldiers” were commonly found in armies during the British Civil Wars. As has been discussed in more detail elsewhere, the age at which it was considered appropriate to take up arms was no less contestable in the Civil Wars than it is now, though legal boundaries were set at eighteen or sometimes sixteen years’ old (Pells forthcoming). Whilst adults tried to prevent those they deemed too young from going to war, this was often resisted by the youths concerned and those perceived to be underage boys even by contemporary standards ended up in the armies on both sides. One such youngster was the duke of Buckingham. He fought in Prince Rupert’s attack on Lichfield Close aged fifteen and later reminded Charles II that he had lost his estate for running away from his studies at Cambridge to fight for Charles’s father “when I was not thought of age sufficient to bear arm[s]” (Yardley 2004; Bell 1849, 2:251).

As well as acting as combatants, children were an essential part of the war effort by performing numerous non-combatant roles in the armies on both sides. I have found evidence for post-boys, trumpeters’ boys, a “boy drummer” and boys who acted as pages, assistants and spies (Kitson and Kitson Clark 1904, 2:164, 178, 180, 188; Rushworth 1645–1648:50; *Mercurius Britanicus* 1645:3; Willis Bund 1920, 2:117; Atherton 2007:83–91; Pafford 1966:75, 77; *Mercurius Britanicus* 1646:11). The evidence for the presence of girls in Civil War armies and the types of roles they may have fulfilled is rather more circumstantial. This is not least because much female labour in early modern Britain generally, not just within armies, was performed without pay. However, there is evidence to suggest that girls were present in Civil War armies and carried out or assisted adult women in tasks such as finding, cooking, and serving food; making, washing, and mending clothes; tending the sick, the infirm, and the wounded (Pells forthcoming).

Historians must be careful of viewing “child soldiers” in Civil War armies as passive victims rather than active survivors. As the example of the duke of Buckingham noted above demonstrates, some youngsters enlisted as combatants seemingly of their own free will. Summerfield noted that modern Western ideals might abhor the concept of child soldiers but in interviews with child-soldiers in many modern conflicts, children have provided rational reasons for joining a militia or army, displayed intense loyalty to their cause, and fought with skill and ingenuity (Summerfield 2000:424–425). Some of these phenomena are apparent in a letter written by the 16-year-old Robert Harley to his elder brother Edward in 1644. Harley was clearly proud of his achievements, describing in detail his role in commanding an out-guard of horse the night before battle of Cheriton and how his troop had been personally selected to lead the first charge in an attack on Newbury (Royal Commission on Historic Manuscripts 1894:107–108).

Likewise, to modern eyes the extensive use of boys and girls for labour in Civil War armies may seem exploitative and likely to result in adverse psychological effects for the children concerned. Yet, as Summerfield discerned, “The child labour that is common in many poverty-stricken parts of the world is deplored in the West because what we understand by a proper childhood appears to have been ‘lost’. But some child labourers do not see themselves as victims, and take pride in what they are doing to keep their family fed” (Summerfield 2000:424–425). A similar willingness on the part of some children to undertake employment and fulfil the tasks assigned to them well may be detected in the Civil Wars. During the storm of Shelford House in 1645, a boy was amongst those captured in the initial stages by the parliamentarian attackers. He was recognized as one who had previously been in the company of John Hutchinson, the parliamentarian governor of Nottingham, until the boy had been captured by the royalists and taken into their service. When in parliamentarian service, the boy had “carried himselfe so stoutly that Captaine Wray beg’d him for a footeboy.” Upon his recapture at Shelford, the boy managed to escape the usual punishment of hanging for defectors by persuading Hutchinson that he could act as a guide to lead the parliamentarian forces to where the royalist defences were unfinished. As a guide, the boy “did such excellent good service, behaving himselfe so stoutly” that Hutchinson himself took him as a footboy (Sutherland 1973:161).

Much of the modern, Western emphasis on the victimhood of child soldiers and the reticence to accord them any agency stems from a discomfort with the notion that the “underage” might be responsible for their participation in a conflict, including their part in atrocities and war crimes, and therefore ought to be brought to justice (Rosen 2007:297). Nevertheless, recent scholarship on child soldiers has argued against a victim–perpetrator dichotomy in relation to child soldiers, recognising that few are exclusively active or passive actors (Derluyn et al. 2015). Whilst some young people appear to have chosen to become combatants, they may not have exercised that choice entirely freely because they are likely to have also been subject to social, cultural, economic or political pressures. In short, “volunteering, compulsory and forced recruitment form parts of a continuum rather than being sharply defined and distinct categories” (Happold 2005:11–12). For youngsters like Robert Harley, family ties and tradition may have played an important part in their enlistment. He was the younger son of the prominent parliamentarian gentleman Sir Robert Harley, and his wife Brilliana, of Brampton Bryan in Herefordshire and he had joined Sir William Waller’s army as a lieutenant in his elder brother’s troop (Roberts 2017). The desire to please his kin may have pressurized as well as inspired Harley into joining Waller’s army and performing well on the battlefield. This phenomenon was perhaps shared by the numerous young gentlemen’s sons who were commissioned into the officer corps of the armies on both sides. For other youngsters, poverty could have been a pressurising factor in their enlistment, even if they took pride in their ability to provide for themselves and their families through military service. For example, the parliamentarian correspondent at the siege of Plymouth in 1643 noted with some discomfort that the parliamentarian defenders had resorted to using ‘poor little boys’ from London (Stoyle 2001:390).

The evidence from the British Civil Wars thus underlines many of the significant points made by Summerfield about the willingness and agency at least displayed by some “child soldiers” but, at the same time, also reminds us that even they may not have been entirely enthusiastic participants and other children likely to have been far from willing to become involved in the armies. Furthermore, children did not have to accompany an army to witness the events or consequences of the Civil Wars first-hand. On separate occasions, a boy gathering peascods and a boy carrying wood were caught in crossfire during the siege of Worcester (Willis Bund 1920, 2:124, 165). When Prince Rupert’s army sacked Leicester in 1645, children were amongst the hundreds that were said to be killed in cold blood, whilst at Birmingham in 1643 his army was said to have left no child alive (Donagan 2008:162; Anon 1643:7). The countess of Derby’s children, Lady Mary and Lady Catherine, were resident with her throughout her defence of their home, Lathom House (Sutherland 1973:515). War displaced numerous children like those who were among the 500 exiles thought to be in London from Devon alone by late 1644 (Stoyle 1996:95). Children were especially susceptible to the diseases brought by troops moving into new areas, which decimated local populations (Purkiss 2006:392). Paul Slack found that amongst the burials recorded for Colyton in Devon, children aged five to nineteen made up twice the percentage of those who died from plague in 1645–46 than the percentage that that age group contributed to deaths in non-plague years (Slack 1985:182).

## Child Psychological Trauma and Theories of Child Psyche in the British Civil Wars

It is impossible to say with certainty if the ever-present prospects of danger, displacement, disease and death may have taken a toll on their young minds of the children who lived through the Civil Wars. When a young boy on his way to join his father in Oxford was stripped and robbed by parliamentarian soldiers, he was said to be “almost distracted with feare” (Donagan 2008:162). However, such an immediate reaction is not enough to suggest that the child suffered from trauma as a result of his experience. Some trauma symptoms commonly emerge a long time after the event that triggered the response (Buck 2012:442–443). Historians searching for evidence of trauma in the past are often confronted by a silence in the sources on the long-term ramifications of a distressing event.

Erin Peters has demonstrated how “Theories about the damaged mind and early ideas of psychiatry” were already circulating in print prior to the Civil Wars. Seventeenth-century authors such as John Sym, Robert Burton, Edward Reynolds, Thomas Povey and Thomas Willis acknowledged that external factors and events had the power to cause significant disorders of the mind (Peters 2018:157–162). However, these authors had much less to say about how or to what extent their theories related to children. For example, Reynolds equated children with the “commonly impotent”, “sick” and “incontinent Persons.” These individuals did experience disorders of the mind, though Reynolds argued that these were most likely to be “*Infirmity, Rashnesse*, and *Mutability* of Mind” because the sufferers were “Temerarious in deprecipitating the Minde, and anticipating the Dictates of Reason which should regulate or restraine them” (Reynolds 1640:177). Burton had likewise maintained that children possessed “no iudgement or counsel,” though he upheld that this made children vulnerable and thus that problems with parents, early childcare and education could all result in adverse psychological conditions amongst children (Burton 1621:81–85, 190–193. Burton even included children in his discussion of “Terrors and affrights causes of melancholy,” narrating the case of a child who saw a grave opened and upon sight of the body “was so troubled in mind, that she could not be comforted, but a little after died” (Burton 1621:194).

This evidence largely corroborates the conclusions drawn by Elizabeth Foyster. She noted that conduct-book writers in the seventeenth century such as William Gouge, John Dod and Robert Cleaver certainly saw children as impressionable, while many other theorists recognized that children were prone to reacting to the adult world around them. Yet, I am hesitant to go so far as Foyster and say that there are few indications that adults at this time perceived “any possibility of psychological damage to a child who witnessed severe or violent incidents of conflict” (Foyster 1999:67). One possible incident where an adult sought to protect children from witnessing violence during the Civil Wars can be found in Richard Gough’s history of the parish of Myddle in Shropshire. Gough, a schoolboy at the time of the conflict, recalled a skirmish that took place in his village. His teacher, Mr Richard Rodericke, “commanded us boys to come into the church, soe that wee could not see the whoale action” (Gough 1875:40). Nevertheless, it is difficult to be sure if Rodericke’s actions were motivated by a desire to protect his charges from physical or psychological harm (or both). Admittedly, I have found no examples to date of seventeenth-century theorists who have discussed psychological conditions resulting specifically from violence or conflict as experienced by children. Nevertheless, as authors such as Burton clearly accepted that distressing experiences could cause challenges to psychological functioning amongst children, it cannot be fully ruled out that seventeenth-century contemporaries held violence and conflict to be one of those potentially damaging distressing experiences for children. A modern, Western concept of the psychologically damaged child as a result of violence and conflict was thus certainly not widespread but unlikely to have been non-existent.

## Contemporary Attempts to Mitigate the Effects of Conflict on Children During the British Civil Wars

Contemporaries in the Civil Wars certainly attempted to limit the effects of the conflict on children through military discipline, punishment, and appeals to the Christian and moral self (Donagan 2008:163). For example, the *Military Orders and Articles* which governed conduct in the king’s armies forbade soldiers from terrorising “any Church-men, aged men, or women, or Maids, or Children unless they first take Arms against them [the soldiers]” (Charles I 1644:19–20). Barbara Donagan has shown how such military regulations were based on a wider European cultural assumption that “It was an offence against the laws of God and nature to kill harmless boys” (Donagan 2008:139). However, children could disqualify themselves from protection if they took up arms (Donagan 2008:163). The understanding that “innocent” youngsters deserved immunity could have a direct impact on children’s experiences of the Civil Wars. Halfway through the storm of Bridgwater in July 1645, the parliamentarian commander-in-chief, Sir Thomas Fairfax, permitted all the women and children to leave the town before the tempo of the assault was increased (Sprigge 1647:71–72). Placing unarmed children amongst the ranks of the harmless to whom protection was afforded in some ways marked an improvement from the medieval period when the children of the aristocracy and gentry were taken prisoner in exchange for ransom. However, this meant that children were subject to less specific military legislation, which could leave them unprotected in situations where they could be used as pawns. In April 1648, the House of Commons ordered the son of the Irish military commander Lord Inchiquin to be sent to the Tower of London (House of Commons 1802:529). This was an attempt to punish Inchiquin, who had just defected back to the king, and pressurize him into releasing English prisoners in Ireland.

Moreover, there were, of course, occasions when frameworks designed to protect children were not rigorously adhered to. This was often as a result of military necessity or the impracticability of enforcement. As Donagan noted, “troops who, contrary to orders, ran amok in hot blood after a hard siege and storm were less likely to be punished harshly than those who killed in cold blood” (Donagan 2008:157). The ferocity of Rupert’s actions at Leicester was excused even by a parliamentarian newsbook as “not any thing to speak of more then what was done in heate” (*The Moderate Intelligencer* 1645:114). That said, although atrocities might be justified by the customs and laws of war, “Public sentiment, in fact, sometimes outstripped theorists” (Donagan 2008:166). In 1648, Fairfax, for whom the welfare of children had been so important at Bridgwater, refused to allow the “starving infants of Colchester” out of the town in order to hasten its surrender. This caused public outrage, even though Fairfax justified his actions under the laws of war (Donagan 2008:162, 339–341). During royalist riots in Norwich the same year, the city armourer, Samuel Cawthorne, was assaulted after he—perhaps accidentally—shot a boy in the melee (Hopper 2018:39).

Although measures put in place to protect children during the Civil Wars sometimes proved inadequate or could be outrightly disregarded, stories of cruelties against children became “propaganda staples of both sides” which were reported with “the confidence that they were outrageous departures from the norms of war and humane conduct” (Donagan 2008:162). The seriousness with which both royalists and parliamentarians took allegations of misconduct against children is clear in the strength of the denials that these allegations were met with the contemporary press. In one instance, the royalist newsbook *Mercurius Aulicus* strenuously denied “one particular wherein the Rebels [the parliamentarians] have slaunder’d us,” namely that royalist forces had “*cruelly murthered men, women, and children at* Wood-house *in Wiltshire.”* Instead, *Mercurius Aulicus* claimed “that there was not one *woman* or *child* in the House; and though the Garrison was taken by assault, yet they all had quarter allowed them” (*Mercurius Aulicus* 1644:1). Similarly, the parliamentarian newsbook *The Moderate Intelligencer* lamented that the inhabitants of Breconshire should be so “bent against the Parliament” that they should believe the stories spoken against the parliamentarian armies that wherever parliament’s forces marched, civilians would “carry away their wives, children, cattle, with what goods they can get, [and] flie into woods” (*The Moderate Intelligencer* 1648:1302). Accusations of atrocities committed against children were intended to portray the enemy as the extremists responsible for the chaos and destruction of the Civil Wars. This then enabled those directing the accusations to portray their own side as the moderates, whose own actions in the conflict were necessary to defeat such extremists.

## The Figure of the Child in Expressions of Contemporary Anxieties During the British Civil Wars

The specific ways in which enemy forces were framed as perpetrators of child cruelties were also used to convey stereotypes of the opposition, which were revealing of contemporaries’ concerns surrounding by the Civil Wars beyond a simple dismay at the bloodshed and devastation wrought by the conflict. Many royalists emphasized the humble origins of some of the parliamentarian political and military leaders, viewing them as little more than dangerous upstarts who disregarded the authority of their betters and aimed at overturning the whole social hierarchy. The ways in which the alleged treatment of Lord Capel’s son at the hands of the parliamentarians during the siege of Colchester was reported in royalist circles drew heavily on this perception. Capel was one of the leaders of the royalist forces besieged in Colchester. His son was captured by two men from the family home at Hadham in Hertfordshire and brought to the parliamentarian camp surrounding the town. Much was made of the fact that he was “very sickly and had scarce rid ever on horseback, or been out of the family,” and that he was “soe ill used that he was forced sometimes to lye in a cabin, and sometimes in a little thachet house, with two soldiers lying by him in straw, and every day was carried around the [siege] works” in order to pressurize his father into surrender (Royal Commission on Historic Manuscripts 1891:45). However, what made these alleged cruelties particularly shocking was “the injury done to the Peers by such an order and acte, he [the boy] being a Peere’s eldest sonne” (Royal Commission on Historic Manuscripts 1891:15).

Conversely, parliamentarians portrayed royalists as the English equivalent of the Catholic armies of the contemporaneous Thirty Years’ War, who purportedly plundered their way across mainland Europe with apparently scant concern for civilian welfare. The king’s nephew, Prince Rupert, was a particular target in the parliamentarian press for reports about pillaging and other offences against civilians, including children. The alleged slaughter of children by forces under his command at Birmingham and Leicester has been noted above. Rupert was the son of the king’s sister, Elizabeth, and Frederick V, elector of the Palatinate (in modern Germany) and sometime king of Bohemia. The prince was born and raised abroad and was a young veteran of the European wars, albeit in Protestant armies. He thus provided a convenient link to the royalist stereotype and a high-profile figure for propagandists to aim at which stopped short of directing criticism at the king himself. In accusing Rupert and his forces of killing children in cold blood, parliamentarian polemicists tapped into widespread fears that England had ‘turned Germany’ and was at the mercy of unrestrained foreign, Catholic and even “heathenish” troops (Roy 1978:128). One pamphlet, which belittled the “titular German Prince” and noted the number of “papists” in his army, laid the blame for the “inhumanity to women and children” committed by Rupert’s forces squarely at the prince’s feet and argued “though we may in a charitable modesty beleeve more hath been imputed to the Prince himselfe then he hath committed, yet we can no way acquit his Souldiers, whose rapacity and barbarisme no Tartars have ever equalled” (G. H. 1642:5, 7).

As this evidence shows, allegations of atrocities against children in the Civil Wars were used especially to designate “otherness” to foreign troops, by marking them out as barbarous and uncivilized. Both sides were forced to recruit mercenaries from abroad into their armies but the presence of foreign troops on English soil caused widespread discomfort (Stoyle 2005:221; Hopper 2012:60–63). Englishmen were equally suspicious of the armies raised in the other nations of Britain and Ireland and signalled their uneasiness about these troops by extending the charges of mistreating children to these forces also. In 1647, a street ballad proclaimed against the Scottish Covenanter army, which had joined the war on the side of parliament in 1644, that “when these Herods shew their cruelty, /The guiltlesse children every one must die” (Anon. 1647a). Reports of atrocities supposedly committed against children by Catholic troops in Ireland were sometimes illustrated by shocking woodcuts. These included throwing children from bridges into rivers to drown, roasting children on spits before fires in front of the bound parents and dashing children’s brains out against walls (Anon. 1647b:[39], [41], [49]). John A. Lynn remarked how these types of images are often reminiscent of portrayals of the Biblical slaughter of the innocents and thus charge the aggressors with inherent wickedness (Lynn 2008:154–155). Furthermore, Diana Gittins asserted that:

Images often convey emotion, trigger associations and memories, and can evoke multiple meanings, some of which may not even be recognized by the viewer. Seeing a picture of a small child, for example, may evoke feelings of empathy or vulnerability, stir unconscious memories of fear or anxiety, or suggest ideals of innocence and a wish to protect. If that child is also designated as representing Jesus, a whole extra body of messages and assumptions imbues it (Gittins 2009:39-40).

However, accusations of atrocities against children as a way of designating “otherness” was a prejudice shared across the nations of seventeenth-century Britain and Ireland. Residual mistrust of the English and the memory of brutal defeat in Tudor rebellions made the Cornish particularly fearful of outside attack. Therefore, Cornish men and women signalled their popular apprehension at the arrival of parliamentarian troops in October 1642 by circulating rumours that their children were in danger (Stoyle 2005:42–43). The English reaction to the other nations’ anxieties for the safety of their children in the presence of English troops highlights the hypocrisy in contemporary attitudes to children in conflict situations. Rumours amongst the Welsh that parliamentarian troops in Monmouthshire in 1644 intended to put children to the sword were dismissed as “strange conceits” with which it was “easy to perswade an irrational and stupid people” (Stoyle 2005:30–31). This can be contrasted with English men and women’s strength of conviction that the tales of child cruelties committed by the Irish were genuine (Stoyle 2005:71). Such contradictions in attitudes could have also have real and dangerous consequences for children in the Civil Wars. Children were amongst the large proportion of camp followers in the king’s army which were believed by the parliamentarians to be Irish. When the victorious parliamentarian army massacred many of the king’s camp followers after the battle of Naseby on 14 June 1645, the incident was justified and dismissed on the basis that the victims were “Irish” and thus the killing spree was retribution for the actions of Catholic troops in Ireland (Stoyle 2005:55, 141). As this incident confirms, the brutalities faced by children during the Civil Wars is not to be denied. It is possible to accept both that some atrocities were committed against children and, at the same time, that reports of atrocities were fabricated, exaggerated and distorted to fulfil political agendas.

Above all, what this discussion reveals is that the manner in which incidents involving children in the Civil Wars were related is resonant with Summerfield’s observation that in many cultures, the turmoil caused by war is often understood and expressed in terms of disruptions to the social and moral order, rather than in a discussion of internal emotions (Summerfield 2000:422). This phenomenon was perhaps clearest in contemporary perceptions of the impact of the conflict on the family and the place of children within it. Christopher Durston described how contemporaries commonly perceived a breakdown in family order and a decline in respect from children to their parents (Durston 1989:130–137). For example, Edward Hyde, earl of Clarendon, claimed that “Parents had no Manner of Authority over their Children, nor children any Obedience or Submission to their Parents; but every one did that which was good in his own Eyes” (Hyde 1761, 2:39). In early modern Britain, the household was regarded as a microcosm of society, in which duty to the head of state was first learnt through duty to the head of the household and the responsibilities of rule learnt through raising children (Charlton 1965:85). Simultaneously, kings were presented as the father of his people. This was an image that Charles I himself cultivated in *Eikon Basilike*, the edited version of his meditations and justifications which was published shortly after his execution. It was thus natural that armed resistance to the king and regicide should provoke speculation about family structures and the relationship between children and parents (Hughes 2012:1). Contemporary anxieties may well have been justified, as there are certainly numerous examples of “grown-up children” who found themselves at odds with their parents during the Civil Wars. Fathers and sons fought in opposing armies. The wider political and religious debates surrounding the conflict likewise caused serious and lasting divisions within some families, with many parent–child relationships turning violent and sometimes broken altogether (Durston 1989:130–137).

However, Durston maintained that tales of children running wild were often “the product of conservative alarm and as a result were frequently overstated” (Durston 1989:134). Indeed, such apprehensions, although heightened by the turmoil of the Civil Wars, were nothing new. Paul Griffiths has demonstrated that sensitivities towards subversive behaviour within families had persisted throughout Tudor and early Stuart Britain (Griffiths 1996:290–350). He argued that early modern households were typically far from the “ordered godly community” portrayed by puritan preachers and contemporary conduct texts, though at the same time he held back from “presenting a bleak view of disorder and misery.” Instead, he portrayed a picture of a “complex situation, which catches both the broad spectrum of emotions inside its four walls, and also the ways in which people could move between different postures ranging from conformity to opposition, and incorporating ‘intermediate’ stances like resignation or indifference.” Ultimately, Griffiths noted that domestic order was not the natural state of affairs but something that required work and something that the authorities often had to pursue to achieve stability (Griffiths 1996:290–291). With this in mind, it is perhaps unsurprising that Durston concluded that in spite of the conflicts between some parents and children during the Civil Wars, overall “relations between parents and children were largely unaffected by wartime dislocation and political and religious discord, the mutual obligations and emotional attachments of parents and children remaining of far greater importance than any considerations of an ideological nature” (Durston 1989:136).

## Conclusion

The evidence from the British Civil Wars presented here is, above all, a useful case study of the continuities and changes in the socio-cultural constructions of children and childhood in warfare over time and place. This study is not intended as an attempt to locate this evidence within a teleological progression from past to present. Instead, the aim was to highlight the diverse nature of both historic and modern child experiences of warfare, and the plethora of ways that these experiences were and are understood and represented by adults. Contemporaries in seventeenth-century Britain and Ireland shared modern Western assumptions that ideally, young people below a certain age should be prevented from joining an army as combatants. However, adults were not in agreement over the precise age below which children should not bear arms, children often resisted adults’ attempts to prevent them going to war, adults could disregard the tender years of new recruits when military necessity dictated and the numerous boys and girls that were essential to the war effort in non-combatant roles were hardly safe from the dangers of war either. This highlights how children negotiate and co-create socio-cultural constructions of childhood with adults, and how such constructions should not be regarded as a simple dichotomy between adults and children that ignores the competing interests within these groups. Furthermore, broader social, cultural, economic and political factors could both influence these constructions and place pressures on young people in differing ways. At the same time, some Civil War youngsters seem to have shared the same willingness to enlist, commitment to a cause and ability to fight well on the battlefield that has been found amongst child soldiers in many parts of the modern world. Amongst the non-combatants, there is little in the surviving evidence to suggest that children in the Civil Wars regarded their army employment as exploitative but like some child labourers in modern conflicts, there is available evidence that they might have taken pride in their endeavours.

This reinforces the view that the extensive use of youngsters in armies is not universally likely to result in adverse psychological effects for the individuals concerned. Indeed, the investigation into seventeenth-century theories of human psyche and psychological trauma presented here warn against a presumed universal understanding of the mind. There is little surviving evidence of children who may have experienced lasting adverse psychological effects as a result of the British Civil Wars, although this is not to say that this did not exist. The writings of contemporary adults on psychological theories seem to have accepted that that external factors and events had the power to cause children significant disorders of the mind, although nowhere is this discussed explicitly in the context of war and violence. Therefore, it is not impossible that contemporaries shared some notion of the modern, Western concept of the psychologically damaged child as a result of violence and conflict, but they certainly did not regard psychological damage in children exposed to conflict as either an inevitable or widespread consequence.

Whilst the writings of adult theorists may not uncover the full picture of how children in the Civil Wars experienced violence and conflict, these could have practical consequences for children in shaping adult behaviour towards children. Adults sought to protect children from the excesses of the conflict and acts of violence against children in the Civil Wars, which could be both restrained and justified by the laws and customs of seventeenth-century warfare, were met with public outrage. Simultaneously, the ways in which incidents of violence against children were presented in print were perhaps most valuable as an insight into the anxieties of contemporary adults. Like many modern cultures, adults in the Civil Wars understood and expressed their anxieties in terms of disruptions to the social and moral order, rather than in a discussion of internal emotions. The figure of the child was often central to these contemporary discourses, which illustrates the ways in which representations of children and violence often become intertwined with political considerations. Politicised discourses had the power to shape children’s experiences of the Civil Wars, the most severe examples of which affected children who took up arms or resided amongst enemy garrisons and armies, or those who were (sometimes mistakenly) held to be of the same ethnicity as enemy forces. These children became tainted by association with the adults around them and were put beyond the bounds of the innocent and protected status usually accorded to the young. By questioning the varied political nature of the representation children and violence in the Civil Wars is therefore not to deny the reality of the brutalities faced by children but a way to gain a deeper understanding of how contemporaries experienced and understood what was, after all, Britain’s bloodiest conflict.
